# Complex Monitoring of Biochemical and Radionuclide Parameters in Patients with Metastatic Renal Cell Carcinoma during Immunotherapy

**DOI:** 10.1155/2017/8549502

**Published:** 2017-10-22

**Authors:** M. S. Sayapina, S. G. Averinova, T. V. Zacharova, A. V. Kashkadaeva, S. V. Shiryaev, M. V. Poluectova, O. A. Vorob'eva

**Affiliations:** ^1^N.N. Blokhin Russian Cancer Research Center, Ministry of Health of Russia, 23 Kashirskoe Shosse, Moscow 115478, Russia; ^2^A. Tsyb Medical Radiological Research Centre, Branch of the National Medical Research Radiological Centre of The Ministry of Health of The Russian Federation, 10 Zhukov St., Obninsk, Kaluga 249036, Russia

## Abstract

**Study Objective:**

To study the effectiveness of complex monitoring of the kidney function, based on biochemical and radionuclide methods in patients with metastatic renal cell carcinoma (mRCC).

**Materials and Methods:**

41 mRCC patients after nephrectomy received nivolumab (*n* = 23) and interferon-*α* (*n* = 18) from 2015 to 2017. At baseline and 2 months after, all patients underwent blood chemistry, urinalysis, Rehberg test, and ELISA to determine serum levels of IL-17A, TGF-*β*, and erythropoietin. The monitoring of the renal function and urodynamics by complex renal scintigraphy (CRS) was used for all patients using a dual-detector gamma camera and simultaneous data recording in 2 projections. The interpretation of CRS data used the original SENS CRS technology.

**Study Results:**

Statistically significant correlations were established between IL-17A, TGF-*β*, and D (excretion rate of 99mTc-technephore from the parenchyma) and Rnfsc (a stable sign of nephrosclerosis), respectively. A significant correlation was established between the parameters of the complex functional monitoring with the prognosis for the risk of renal failure (RF) and efficacy of immunotherapy in mRCC.

**Conclusions:**

All mRCC patients after nephrectomy were recommended to undergo biochemical monitoring with inclusion of TGF-*β* and IL-17A, as well as radionuclide monitoring (CRS) to determine the RF risk at an early stage.

## 1. Introduction

Literature describe cases of proteinuria and irreversible renal failure during immunotherapy and targeted therapy in patients with mRCC, resulting in a dose reduction and/or drug withdrawal, which can affect the objective response [1–4]. All patients with mRCC after nephrectomy should be assigned to a high risk group for developing chronic kidney disease (CKD).

Until recently, clearance of endogenous creatinine was the most widely used method for determining GFR in clinical practice. However, in moderate to severe renal insufficiency, GFR values calculated from the endogenous creatinine clearance are significantly overestimated, since creatinine is secreted by the proximal tubule in the settings of renal failure and uremia [5, 6].

In order to estimate GFR, formulas such as MDRD (Modification of Diet in Renal Disease), Cockcroft-Gault, CKD-EPI (Chronic Kidney Disease Epidemiology Collaboration), and MCQ (Mayo Clinic Quadratic) are widely put into practice. Currently, there is evidence allowing suggesting that the screening diagnosis of CKD should be based on only a simultaneous estimation of GFR and albuminuria/proteinuria, which allows estimation of the prognosis and risk of cardiovascular events [7].

The KDIGO (Kidney Disease: Improving Global Outcomes) classification, as is the case with RIFLE (risk, injury, failure, loss, and end-stage kidney disease) and AKIN (acute kidney injury network), based on serum creatinine levels and the volume of diuresis, allow timely diagnosing acute kidney damage and have a prognostic value, but do not allow to take into account causes of kidney damage and, therefore, do not always help determine preventive and treatment tactics [8, 9]. In this regard, the search for the most accurate biomarkers that would allow for early diagnosis of CKD and establishing the cause of its development is of current concern. At the moment, a number of biomarkers associated with nephrotoxicity are known, but FDA (Food and Drug Administration) and EMEA (European Medicines Agency) have approved only KIM-1 (kidney injury molecule-1), albumin, total protein, *β*2-microglobulin, cystatin C, clusterin, and TFF3 (trefoil factor 3) for routine use in practice [10, 11].

The rate of progression of chronic renal failure (CRF) is noted to be proportional to the rate of the renal parenchyma sclerosis, a fundamental component of the CRF pathogenesis [6]. Transforming growth factor-*β* (TGF-*β*) plays the most prominent role in this process. The signaling pathways of this growth factor (Smad, p38, Erk1/2, PI3K, JNK, etc.) can cause glomerulosclerosis and tubulointerstitial fibrosis via multiple pathological processes [12]. An increase in TGF-*β* activity stimulates the cell proliferation and accumulation of extracellular matrix (ECM) components, such as collagen types I, III, and IV, laminin, and cellular and plasma forms of fibronectin, which contributes to the development of glomerulosclerosis [12].

Hemodynamic effects of glomerular hypertrophy caused by the loss of the renal mass are closely related to the mechanisms of persisting kidney inflammation and fibrosis via interaction of angiotensin II, TGF-*β*, and other growth factors. In addition to hemodynamic effects, primarily a systemic vasoconstrictor action also transmitted to the glomerular capillaries. So angiotensin II has so-called inherent nonhemodynamic effects, including the ability to induce endothelial dysfunction, as well as to enhance local renal expression of TGF-*β*. A nonhemodynamic component of the angiotensin II effect plays the main role in aggravation of proteinuria, which is why the drugs that block its formation (ACE inhibitors) or interaction with type 1 receptors (angiotensin II receptor blockers) have prominent antiproteinuric properties [6].

Of interest is investigation of IL-17 concentrations in the serum, having strong proinflammatory properties and inducing severe autoimmune pathology, including nephritis [13, 14].

**Figure 1:**
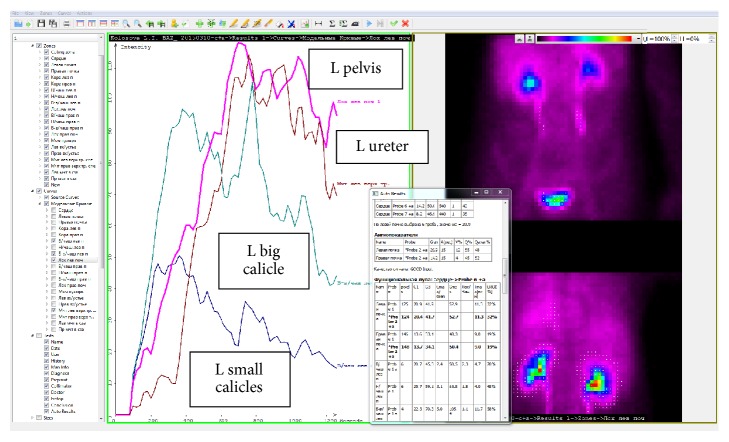
An automated workstation implementing the SENS-CRS technology on a personal computer by processing CRS DICOM (Digital Imaging and Communications in Medicine) files generated by a modern dual-detector gamma camera. In the upper band a set of tool icons is used in the workstation in the analysis of data of functional radionuclide studies of the kidneys. In the left part there is a set of zones of interest (an interphase in Russian language), selected on scintigraphic images of the kidneys and urinary tract according to the SENS-CRS technology. The curves obtained from the automated workstation represent the dynamics of the concentration of urine labeled with a nephrotropic radiopharmaceutical agent (99mTc-MAG3) in the structures of the left (L) kidney: the group of small upper calicles, the big upper calicles, the pelvis, and the ureter (delay of labeled urine in the middle third). Scintigrams on the right: top, image of the urinary system in the front projection; bottom, in the back projection. At the top there is a color scale (the red color corresponds to the maximum score on scintigrams). In a separate window (in the center): results of a quantitative estimation on original algorithms of function parameters of urinary system are shown.

In recent years, glomerulotropic radiopharmaceuticals labeled with radioisotopes are widely used as marker substances allowing determining GFR [15]. The diagnostic value of renal purification from nephrotropic substances (^99m^Tc-MAG_3_, ^123^I-hippuran, and ^99m^Tc-DTPA) closely correlates with inulin clearance [16–18]. However, GFR studies using radioactive isotopes are used only if specialized radiological laboratories are available [19, 20]. In this regard, the Laboratory for Radioisotope Diagnosis of Russian N.N. Blokhin Cancer Research Center, a federal state-funded scientific institution, has developed the systemic examination of nephrourological status based on complex renoscintigraphy (SENS-CRS) and has been using it in pediatric and adult clinical practice for more than 15 years [19, 21–23]. SENS-CRS is a high technology implemented in the development of an automated workplace (AWP) for a radionephrologist (Project Manager A.P. Alekhin) (Figure 1).

SENS-CRS is designed for a rapid assessment of the functional reserves of the urinary system and the risk of renal failure. The CRS method provides not only the monitoring of the concentration levels in the parenchyma, but also early detection of relative stagnation in the parenchyma, its edema, urine stasis in the departments of the pyelocaliceal system (PCS), and lower urinary tract, that is, at all functional structural levels. Biochemical parameters of the kidney function, such as serum creatinine and urea, reflect quite gross morphologic alterations in the renal parenchyma and become diagnostically significant when 50 to 70% of active nephron mass (ANM) have already become dysfunctional [19].

When planning this study, it was assumed that complex monitoring of the renal function based on biochemical and RN methods will allow diagnosis of the risk factors for RF at an early stage, differentiate the structural kidney damage from the functional one, determine their relationship with immunotherapy toxicity and efficacy in patients with mRCC, and timely prescribe concomitant therapy.

## 2. Materials and Methods

This study included 41 mRCC patients after nephrectomy within the period from 2015 to 2017. 18 patients were treated with interferon (IFN-*α*) and 23 patients were treated with nivolumab (as part of the BMS expanded access program). Of 18 patients treated with IFN-*α*, 16 patients (88.8%) received it as the first line therapy. The median age was 56 years. Before (within a week) and during the treatment (every 2 months), all patients underwent blood chemistry, urinalysis, and Rehberg test.

The serum levels of proteins studied were determined according to the standard procedure prior to the treatment (within a week) and 2 months after. The serum was obtained after blood centrifugation at 3000 rpm, 4°C for 10 min using RS-6 model centrifuge (“Technocom”, Russia). 300–400 *μ*L of serum were dispensed in 2 plastic tubes and stored at −80°С until the analysis. ELISA tests for IL-17 (eBioscience, USA), TGF-*β*1 (eBioscience, USA), and EPO (erythropoietin) (Biomerica, USA) were performed using standard kits for direct immunoassay according to manufacturer's instructions.

Complex renoscintigraphy (CRS) was carried out on a dual-detector gamma camera (E-com, Siemens) with simultaneous recording in 2 projections, which allowed studying the entire renal clearance system, starting with the heart blood flow and ending with the bladder. Diagnostic simulation of renal clearance from nephrotropic substances begins with intravenous administration of ^99m^Tc-technephore. ^99m^Tc-technephore, a Russian product from the group of bisphosphonates, has hemodynamics of a glomerulotropic product, concentrating mainly in the nephrons via filtration, with partial involvement of secretion. The visualization quality (even with a weak kidney function) of ^99m^Tc-technephore is comparable to that of tubulotropic ^99m^Tc-MAG_3_ (mercaptoacetyltriglycine) and ^123^I-hippuran, significantly outperforming conventional glomerulotropic ^99m^Tc-DTPA (diethylenetriaminepentaacetic acid) [21]. The data registered in 2 projections is processed based on 2-phase registration: the first step is a 21-minute (1 min, angiophase) basic test with administration of the labeled substance; the second phase is delayed (after a 25-minute break) 21-minute examination (sometimes a 7-minute test) without administration of RP, but with administration of small amounts of water (200–300 ml) and/or an antispasmodic (less frequently diuretic). The bladder emptying by the patient before the baseline test and examination is a mandatory functional test. Complex renoscintigraphy allowed achieving the lowest radiation doses for patients and staff. When CRS is performed, adults are administered intravenously 74 MBq of ^99m^Tc-technephore (an effective equivalent dose of 0.6 mSv), less frequently ^99m^Tc-technephore; children are given a radiopharmaceutical based on their age and body weight. When kidneys and bones are investigated on the same day, adults are given 370 to 555 MBq of ^99m^Tc-technephore (an effective equivalent dose of 3.0–4.5 mSv). The interpretation of CRS data used a concentration-rate model of urinary excretion and the original SENS CRS technology developed in the laboratory of radioisotope diagnostics, Russian N.N. Blokhin Cancer Research Center. The level of concentration of both glomerulo- and tubulotropic radiopharmaceuticals in the parenchyma is proved to be a well reproducible measure of the kidney concentration function [21, 24]. The statistical analysis of the results was performed using software Statistica 13.0 with the Spearman nonparametric method (*R*_Sp_ is a correlation coefficient; the result was considered insignificant at *p* ≥ 0.05).

## 3. Study Results and Discussion

This study included 41 mRCC patients after nephrectomy within the period from 2015 to 2017; 18 patients were treated with IFN-*α* and 23 patients were treated with nivolumab. Of 18 patients treated with IFN-*α*, 16 patients (88.8%) received it as the first line therapy. Twelve (12) patients (52%) and 11 patients (48%) in the nivolumab group received nivolumab as their second line and third or further line therapy. Thus, given the large number of previous lines of therapy, the nivolumab group had a higher risk of developing tubulointerstitial nephritis (TIN). Thus, the incidence of stage-3 CKD at the time of treatment was 35% in the nivolumab group and 17% in the IFN-*α* group.

Inflammation of the renal tubulointerstitium is always clinically characterized by impairment of the renal concentration function and often renal filtration function. Renal glomeruli can be abnormal, but abnormalities have a secondary nature [7]. As a result, the amount of the radiopharmaceutical concentrates reduced during a radionuclide study, which determines the fundamental premise of the complex renography (CR), the “concentration function” as a total result of all processes in the renal parenchyma [25]. In SENS-CRS, an algorithm was developed that determines the level of compensation and the risk of destabilization of the total renal function in gradations of FSS (Functional Systems Scores), the total prognostic index of the urinary system functional state, and stability. The relationship between different degrees of clinical parameter gradations—CKD according to KDOQI (Kidney Disease Outcomes Quality Initiative) and KDIGO—and RN parameter—total prognostic index (FSS) according to CRS findings with nephrotropic radiopharmaceuticals (^99m^Tc-technephore, ^99m^Tc-DTPA, ^99m^Tc-technemag, ^99m^Tc-MAG3)—are presented in Table 1.

The grades of the FSS index are the same for the above radiopharmaceuticals (the differences between these drugs are taken into account within the SENS-CRS algorithm). A single concentration-rate approach to the study of kidney function and urodynamics of the urinary tract with different nephrotropic radiopharmaceuticals is described in detail in the publication [21, 26].

The rate of irreversible deterioration of the kidney function in most variants of TIN is much slower than that in other chronic progressive nephropathies. In our study, only 1 patient (2.4%) developed acute renal failure (ARF) after 2 injections of nivolumab. It should be noted that this was the only patient who initially had the lowest RN estimate for the total urinary system function, FSS = 3b (significantly reduced). The cause elimination is crucial in the management of patients with TIN. In this particular case, the patient probably developed tubular necrosis as early as during previous therapy with everolimus (for 2 years); however, at the time of the initiation of nivolumab treatment, GFR, calculated by the MDRD formula, was 41 ml/min. Due to the development of acute renal failure, nivolumab treatment was discontinued. The patient was switched to dialysis.

The presence of confounding factors that can increase the severity of renal disease should be taken into account: chronic heart failure; type 2 diabetes; impaired uric acid metabolism. Elderly patients can have a combination of several forms of renal disease (“multimorbidity”), such as analgesic, urate, and diabetic nephropathy, as well as ischemic renal disease (IRD) and chronic pyelonephritis [6]. In our study, hypertension was observed in 15 patients (36.5%), type 2 diabetes in 4 patients (9.7%), urinary infection in 4 patients (9.7%), urolithiasis in 2 patients (4.8%), and obesity in 2 patients (4.8%).

### 3.1. Analysis of Biochemical and Radionuclide Parameters during Immunotherapy

The analysis of blood chemistry parameters (creatinine, urea), daily Rehberg test, total protein in the urine, and total and partial RN parameters established their relationship, which confirmed the diagnostic value of both methods—biochemical and CRS (Table 2). We analyzed 97 clinical cases (during the monitoring) constituting the group of patients after nephrectomy during therapy with IFN-*α* and nivolumab.

**Figure 2:**
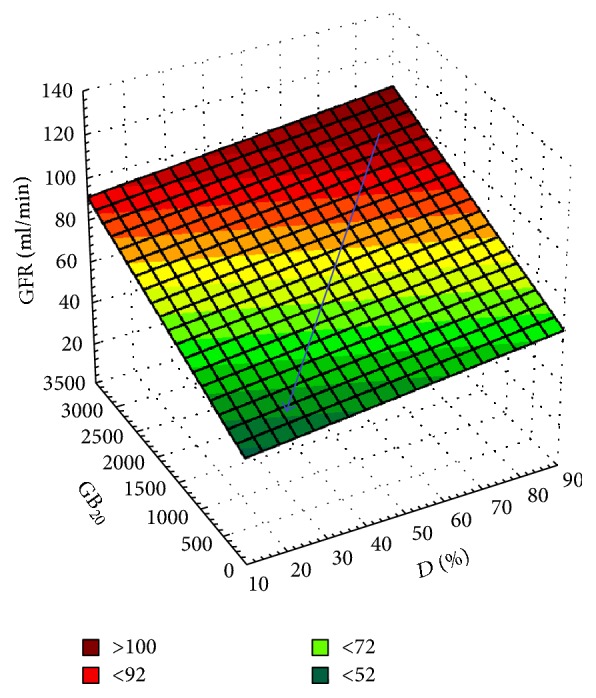
A linearly smoothed 3D-surface showing the relationship between GFR in the Rehberg test and RN parameters, *D* and GB_20_. The blue arrow shows the development of CTIN (chronic tubulointerstitial nephritis) and an increase in the risk of RF, followed by a decrease in the urine excretion rate in the renal parenchyma (*D*), the level of concentration of labeled urine that entered in the bladder within 20 minutes of the basic CSR test (GB_20_), and GFR estimated by the Rehberg method (*R*_Sp_ = +0.4, *p* < 0.01).

There was also a statistically significant relationship between the total GFR based on the 24-hour Rehberg test and RN parameters, *D*, % (RP excretion rate from the parenchyma at the “cortex-medulla” level) and GB_20_ (a 20-minute level of RP concentration in the urinary bladder in the basic CRS test) (Figure 2).

**Figure 3:**
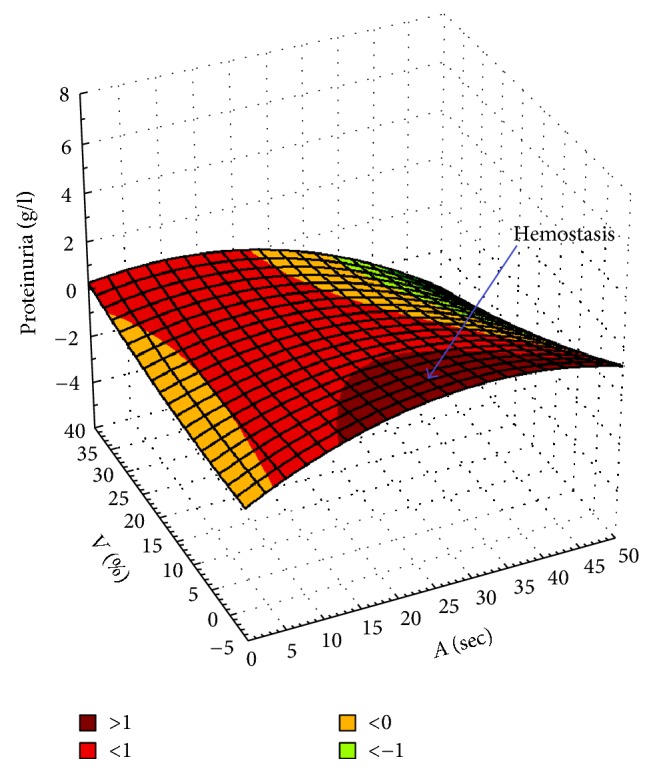
The 3D surface (spline-smoothing) showing a relationship between proteinuria and hemodynamic parameters of kidney parenchyma, *A* [s] and *V* [%]. The blue arrow points to an area corresponding to pronounced hemostasis (parameters *A*-*V*) observed with an increase in proteinuria with the development of CTIN and a possible urinary tract infection (UTI).

An increase in proteinuria up to the nephrotic level is determined primarily by the loss of the selectivity of the glomerular basement membrane and progressive podocyte dysfunction. This disorder is also accompanied by inappropriate activation of the renin-angiotensin-aldosterone system, typical of many variants of nephrotic syndrome and resulting in sodium retention and osmotically bound water aggravating the edema in addition to the developed resistance of respective nephron segments to natriuretic peptides [7]. Hypercoagulability typical for nephrotic syndrome is determined primarily by the activation of serum and endothelial hemostasis, which results in an increased risk of venous thrombosis and thromboembolism. This phenomenon is reflected during CRS, demonstrating the relationship between proteinuria and hemodynamic parameters of the renal parenchyma, *A* [s] (a perfusion rate of renal blood labeled with RP, an arterial index of renal parenchyma) and *V* [%] (a clearance rate of RP-labeled blood via the renal venous system, a venous index of renal parenchyma) (Figure 3).

**Figure 4:**
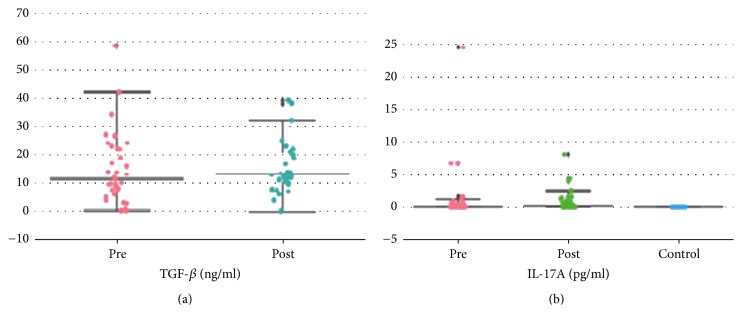
(a) TGF-*β* content in the serum of 40 mRCC patients before the initiation of immunotherapy with the inclusion of INF-*α* and nivolumab and after 2 months. (b) IL-17A content in the serum of 40 mRCC patients before the initiation of immunotherapy with the inclusion of INF-*α* and nivolumab and after 2 months (in comparison with the parameters of the control group, *n* = 10).

With regard to the search of diagnostic markers of early stages of renal dysfunction, we analyzed transforming growth factor-*β* (TGF-*β*) as a factor of renal parenchyma sclerosis and IL-17 having strong proinflammatory properties and inducing severe autoimmune pathology, including nephritis, in the serum of 40 patients with mRCC prior to immunotherapy (IFN and nivolumab) and 2 months after. Against the backdrop of immunotherapy with the inclusion of INF-*α* and nivolumab, there was a significant increase in IL-17A from 0 ± 4.29 (median ± SD) to 0.166 ± 1.714 pg/ml (*p* < 0.0005) and a trend toward TGF-*β* growth from 11.3 ± 12.4 to 13 ± 10.1 ng/ml (*p* = 0.1) (Figure 4). The study was able to compare TGF-*β* and IL-17 values with RN parameters, Rnfss, a stable sign of nephrosclerosis (presumably sclerosis of interlobar renal arteries), and *D*, an excretion rate of ^99m^Tc-technephore from the parenchyma (at the “cortex-medulla” level), respectively (Table 3).

Thus, an increase in TGF-*β* concentrations correlates with Rnfsc during CRS, which confirms the diagnostic value of Rnfsc as a visual radionuclide sign of “nephrosclerosis”, whose assessment was carried out according to the grading specified in Table 4. Moreover, in the nivolumab group, nephrosclerosis was much more pronounced than in the IFN-*α* group, which may be due to a larger number of previous lines of targeted therapy.

At the same time, an increased IL-17 level both prior to immunotherapy and 2 months after corresponded to a decrease in the RP excretion rate from the parenchyma (*D*) due to increasing interstitial edema, confirming the importance of cytokine IL-17 in the pathogenesis of autoimmune nephritis.

It should be noted that these biochemical and RN markers not only allow establishing impairment of the renal function at an early stage, but also differentiating the cause of this disease, nephrosclerosis or an autoimmune condition, which is extremely important in determining the treatment strategy for CKD.

**Figure 5:**
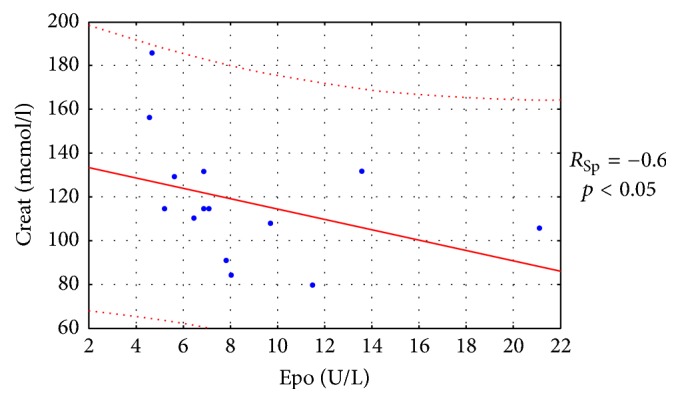
Correlation between erythropoietin (Epo) and creatinine (Creat) in the serum of mRCC patients prior to the treatment with IFN-*α* (*n* = 15).

Also, in the IFN-*α* group, endogenous erythropoietin levels were evaluated by ELISA in 15 mRCC patients after nephrectomy prior to the treatment. The findings had a significant correlation with creatinine levels before the treatment (*R*_Sp_ = −0.6, *p* < 0.05). Thus, reduced erythropoietin levels correlated with increased creatinine concentrations, which is consistent with the pathophysiological basis of the renal function (Figure 5).

In the SENS-CRS technology, radionuclide (CRS with 99mTc-technefor or 99mTc-MAG3, rarely with 99mTc-DTPA) images of the kidneys in the gray scale are obtained using a 64 × 64 matrix, chosen to minimize the dose of the radiopharmaceutical administered and the patient's radiation load.

### 3.2. The Prognostic Value of Laboratory Diagnostic and Biochemical Parameters

At the first stage, we evaluated the effect of RN and biochemical parameters on the RF risk estimated based on an increase in creatinine and urea levels during immunotherapy with IFN-*α* and nivolumab. The data of the nonparametric correlation analysis for parameters potentially significant for the prognosis of RN risks are presented in Table 5.

**Figure 6:**
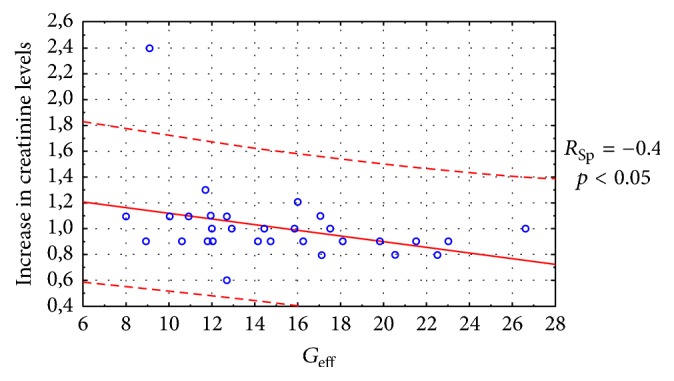
Correlation between a 1.2-fold increase in creatinine levels (±0.4) during immunotherapy and *G*_eff_ prior to the treatment initiation.

**Figure 7:**
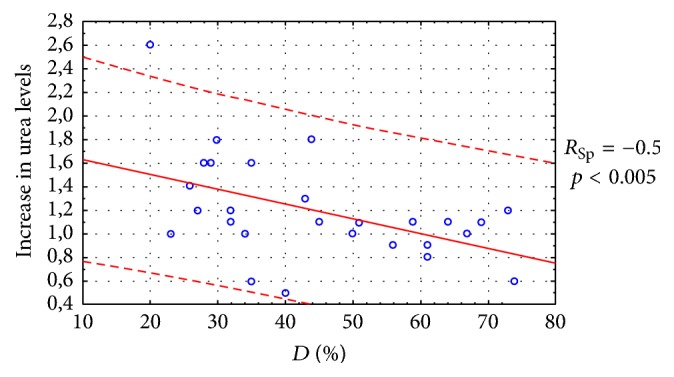
Correlation between a 1.6-fold increase in urea levels (±0.6) during immunotherapy and *D*, % prior to the treatment initiation.

Statistically significant correlations are established between an increase in creatinine levels and IL-17, FSS, *G*_eff_, and Rnfss (Figure 6) and an increase in urea and protein levels in the urine, IL-17, *D*, *T*_ev_, and *T*_pelv_ (Figure 7). Thus, the initial IL-17 value in the serum can be an early predictor for RF development in CKD during immunotherapy, whereas serum creatinine and urea levels gave no statistically significant results in terms of the RF prognosis.

Also, a correlation was observed between the RN sign of nephrosclerosis (Rnfss) and an increase in creatinine levels. Thus, despite the lack of a statistically significant correlation between serum TGF-*β*_1_ and an increase in creatinine levels, but given the correlation between TGF-*β*_1_ and Rnfss (*p* < 0.005), TGF-*β*_1_ can be considered as a relative risk factor for RF development during immunotherapy in patients with mRCC who underwent nephrectomy.

At the next stage, we evaluated the effect of RN and biochemical parameters on the effectiveness of immunotherapy estimated according to the RECIST criteria (Table 6).

As it turned out, the CRS findings have prognostic significance in relation to not only the RF risk, but also the efficacy of immunotherapy in patients with mRCC after nephrectomy. It should be noted that most of correlations were obtained due to statistically significant relationships in the IFN-*α* group. Thus, patients with mRCC with better kidney function parameters have a better prognosis regarding the efficacy of immunotherapy.

## 4. Conclusion

Thus, immunotherapy has no pronounced nephrotoxicity. All mRCC patients after nephrectomy were recommended, prior to the treatment initiation, to undergo biochemical monitoring with inclusion of TGF-*β*_1_ and IL-17, as well as radionuclide monitoring (SENS-CRS) to determine the RF risk at an early stage and to establish prognosis for the underlying disease and timely adjust the treatment in order to improve their response to immunotherapy.

## Figures and Tables

**Table 1:** Relationship between CKD and FSS gradation: a radionuclide estimate for the total renal function.

CKD stage	Renal function characteristic	GFR level (mL/min/1.73 m^2^)	FSS characteristic	FSS index gradation
1	High and optimal	>90	*Status 1* high level slightly decreased (stable or conditionally stable compensation)	1a
2	Slightly decreased	60–89		1b

3а	Slightly decreased	45–59	*Status 2* moderately decreased (conditionally stable compensation, transitionally unsustainable level of compensation)	2a
3b	Significantly decreased	30–44		2b

4	Severely decreased	15–29	*Status 3* moderately to severely decreased (unstable compensation or decompensation)	3a 3b

5	End-stage renal failure	<15	Decompensation	4

**Table 2:** The relationship between biochemical and radionuclide parameters in the monitoring of the urinary system function.

*N* = 97	Plasma creatinine level (112.3 ± 24.4 mcmol/L)	Plasma urea level (7.3 ± 2.4 mmol/L)
*RK*, compensation level	*R*_*Sp*_ ** = ** *+0.3*, *p* < 0.01	Trend → *R*_Sp_ = *+0.2* (*p* > 0.05)

*FSS*, a total prognostic index for the urinary system	*R*_*Sp*_ ** = ** *+0.3*, *p* < 0.005	*p* > 0.05

*G*_*ren*_, a measured level of ^99m^Tc-technephore in the renal parenchyma	*R*_*Sp*_ ** = ** *−0.3*, *p* < 0.001	*R*_*Sp*_ ** = ** *−0.3*, *p* < 0.01

*A* *[s]*, an arterial index of renal parenchyma	*p* > 0.05	*p* > 0.05

*IF*_*ost*_, a speed index of the ureteral orifice (during examination)	*R*_*Sp*_ ** = ** *−0.3*, *p* < 0.01	*p* > 0.05

**Table 3:** Relationship between ELISA findings and RN parameters.

*N* = 40 (before the treatment initiation)	TGF-*β*		IL-17	
	Before therapy	In 2 months	Before therapy	In 2 months
*D, %*, an excretion rate of ^99m^Tc-technephore from the parenchyma (at the “cortex-medulla” level)	Trend → *R*_Sp_ = *−0.3* (*p* = 0.08)	Trend → *R*_Sp_ = *−0.3* (*p* = 0.06)	*R*_*Sp*_ * = −0.3,* *p* < 0.05	*R*_*Sp*_ * = −0.5,* *p* < 0.005

*Rnfsс*, a visual sign of nephrosclerosis (sclerosis of interlobar renal arteries)	*R*_*Sp*_ * = +0.5,* *p* < 0.005	*p* > 0.05	*p* > 0.05	*R*_*Sp*_ * = −0.3,* *p* < 0.05 (polyuria?)

**Table 4:** The rating scale of an RN visual sign of “nephrosclerosis” in points.

*Rnfsc*, a visual sign of nephrosclerosis	Points	Typical scintigrams			
		*Left kidney* Basic test (left), examination (right)		*Right kidney* Basic test (left), examination (right)	
None (?)	0	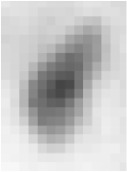	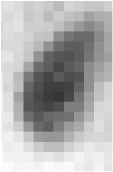	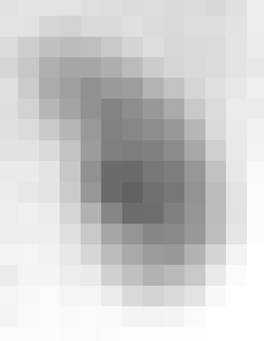	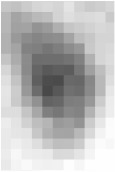

Contradictory picture of initial changes	0.5	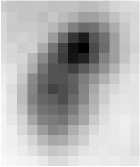	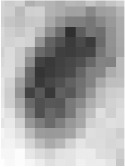	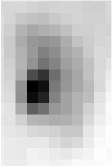	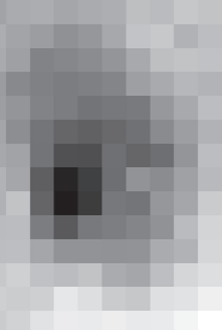

Unsure sign of “nephrosclerosis”	1	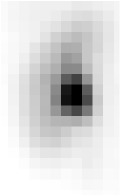	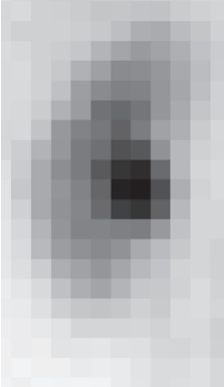	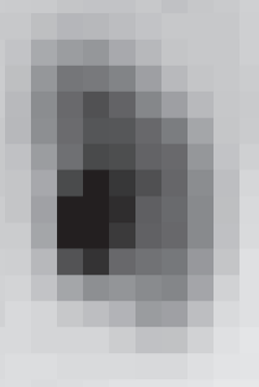	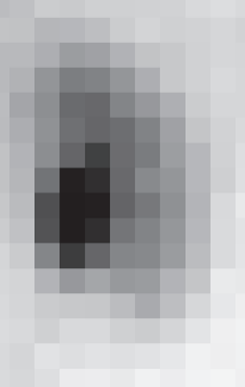

Picture of irreversible “nephrosclerosis”	2	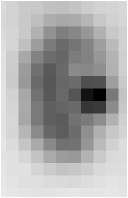	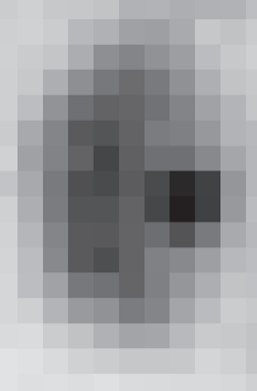	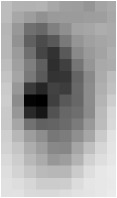	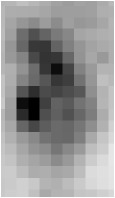

**Table 5:** An effect of biochemical and radionuclide parameters on the prognosis for RF risk during immunotherapy.

Before initiation of immunotherapy	A 1.2-fold increase in plasma creatinine level (±0.4)	A 1.6-fold increase in plasma urea level (±0.6)
*Parameters of laboratory blood and urine tests (n* = 40)		
*Plasma creatinine level*	*p* > 0.05	*p* > 0.05
*Plasma urea level*	*p* > 0.05	*p* > 0.05
*Protein* in urine	Trend → *R*_Sp_ = **+***0.3* (*p* = 0.06)	*R*_*Sp*_ = *+0.5*, *p* < 0.05
*Plasma TGF-β*_1_	Trend → *R*_Sp_ = **+***0.3* (*p* = 0.08)	Trend → *R*_Sp_ = **+***0.3* (*p* = 0.08)
*Plasma IL-17*	*R*_*Sp*_ = *+0.4*, *p* < 0.05	*R*_*Sp*_ = *+0.4*, *p* < 0.05

*Parameters of complex renoscintigraphy with * ^99*m*^ *Tc*-*technephore (n* = 31)		
*FSS*, a total prognostic index for the urinary system	*R*_*Sp*_ = *+0.4*, *p* < 0.05	*p* > 0.05
*G*_*eff*_, an effective index of the renal concentration function	*R*_*Sp*_ = *−0.4*, *p* < 0.05	*p* > 0.05
*D, %*, an excretion rate of ^99m^Tc-technephore from parenchyma	*p* > 0.05	*R*_*Sp*_ = *−0.5*, *p* < 0.005
*T*_*ev*_ * [min]*, the time of start of excretion of the labeled urine from the pyelocaliceal system	*R*_*Sp*_ = *+0.4*, *p* < 0.05	*R*_*Sp*_ = *+0.5*, *p* < 0.01
*T*_*ev*_ * [min],* the time of start of excretion of the labeled urine from the renal pelvis	Trend → *R*_Sp_ = **+***0.3* (*p* = 0.06)	*R*_*Sp*_ = *+0.5*, *p* < 0.005
*Rnfsc*, a visual sign of nephrosclerosis	*R*_*Sp*_ = *+0.4*, *p* < 0.05	*p* > 0.05

**Table 6:** An effect of biochemical and RN parameters on the immunotherapy effectiveness.

Before initiation of immunotherapy (*n* = 40)	Treatment effect
*Parameters of laboratory blood and urine tests*	
Plasma creatinine level Plasma urea level Protein in urine TGF-*β*_1_ IL-17	*p* > 0.05

*Parameters of complex renoscintigraphy with * ^99*m*^ *Tc-technephore*	
*FSS*, total prognostic index of the urinary system	*R*_*Sp*_ * = −0.4*, *p* < 0.05
*G*_*eff*_, an effective index of the renal concentration function	*R*_*Sp*_ * = +0.3*, *p* < 0.05
*T*_*ev*_ * [min]*, the time of start of excretion of the labeled urine from the pyelocaliceal system	*R*_*Sp*_ * = −0.4*, *p* < 0.05
*T*_*ev*_ * [min]*, the time of start of excretion of the labeled urine from the renal pelvis	*R*_*Sp*_ * = −0.3*, *p* < 0.05
